# CCMT: Dataset for crop pest and disease detection

**DOI:** 10.1016/j.dib.2023.109306

**Published:** 2023-06-12

**Authors:** Patrick Kwabena Mensah, Vivian Akoto-Adjepong, Kwabena Adu, Mighty Abra Ayidzoe, Elvis Asare Bediako, Owusu Nyarko-Boateng, Samuel Boateng, Esther Fobi Donkor, Faiza Umar Bawah, Nicodemus Songose Awarayi, Peter Nimbe, Isaac Kofi Nti, Muntala Abdulai, Remember Roger Adjei, Michael Opoku, Suweidu Abdulai, Fred Amu-Mensah

**Affiliations:** a Department of Computer Science and Informatics, University Energy and Natural Resources, Sunyani, Ghana; b Department of Horticulture and Crop Production, University Energy and Natural Resources, Sunyani, Ghana; c Ghana Developing Communities Association, Ghana; d Council for Scientific and Industrial Research, Water Research Institute, Accra, Ghana

**Keywords:** Artificial Intelligence, Dataset: Deep learning, Classification, Pest detection: Plant disease detection

## Abstract

Artificial Intelligence (AI) has been evident in the agricultural sector recently. The objective of AI in agriculture is to control crop pests/diseases, reduce cost, and improve crop yield. In developing countries, the agriculture sector faces numerous challenges in the form of knowledge gap between farmers and technology, disease and pest infestation, lack of storage facilities, among others. In order to resolve some of these challenges, this paper presents crop pests/disease datasets sourced from local farms in Ghana. The dataset is presented in two folds; the raw images which consists of 24,881 images (6,549-Cashew, 7,508-Cassava, 5,389-Maize, and 5,435-Tomato) and augmented images which is further split into train and test sets. The latter consists of 102,976 images (25,811-Cashew, 26,330-Cassava, 23,657-Maize, and 27,178-Tomato), categorized into 22 classes. All images are de-identified, validated by expert plant virologists, and freely available for use by the research community.

**Specifications Table**

**Table:** 

Subject	Machine Learning / Deep Learning
Specific subject area	Crop pest/disease detection
Type of data	Plant pest and disease images
How the data were acquired	The Plant pest and disease images were collected by taking images using a high-resolution camera device. Table 1 shows a description of the camera used to collect the dataset.
Data format	Raw, Annotated Augmented
Description of data collection	The Crop pest and disease datasets were collected using a high-resolution camera device. The original .jpg images were in varied dimensions, namely; (400 × 400), (487 × 1080), (1080 × 518), (3024 × 4032), and (4032 × 3024). There are 22 classes in total. Cashew has 5 classes: anthracnose, gummosis, healthy, leaf miner, and red rust. Cassava has 5 classes: bacterial blight, brown spot, green mite, healthy, and mosaic. Maize has 7 classes: fall armyworm, grasshopper, healthy, leaf beetle, leaf blight, leaf spot, and streak virus. Tomato also has 5 classes: healthy, leaf blight, leaf curl, septoria leaf spot, and verticillium wilt. The images were captured under various conditions and with different backgrounds such as white, dark, illuminated, and real backgrounds.
Data source location	University of Energy and Natural Resources P.O. Box 214, Sunyani – Ghana Website: https//www.uenr.edu.gh African Technology Policy Society Network 8^th^ Floor – The Chancery – Valley Road - Nairobi P.O. Box 10081-00100, Nairobi, Kenya Website: http://www.atpsnet.org
Data accessibility	Repository name: Dataset for Crop Pest and Disease Detection Data identification number(doi): 10.17632/bwh3zbpkpv.1 Direct URL to data: https://data.mendeley.com/datasets/bwh3zbpkpv

## Value of the Data

• The dataset is comprehensive and consists of 102,976 high-quality images of four crops with 22 different classes, respectively cashew (5 classes), cassava (5 classes), maize (7 classes), and tomato (5 classes).
• The dataset consists of plant leaves, pests, fruits and images of sick parts of cashew, cassava, maize, and tomato.
• This dataset is useful for building applications for pest and disease classification, detection, and recognition
• This dataset is useful for training, testing, and validating plant pests and diseases or for classification and identification models.
• The dataset will play an important role in the plant pest and disease identification.
• The dataset will help build an application for plant pest and disease classification, identification, and detection that can be used by farmers, agricultural extension officers, Ministry of Food and Agriculture (MoFA), and various agencies.

## 1 Objective

Human society needs to increase food production by an estimated 75% by 2050 to feed an expected population size that is predicted to be over 9.7 billion people [15,16]. Currently, infectious diseases and pests reduce yield by an average of 38% with many farmers in the developing world experiencing yield losses as high as 99%. The increase in the usage of smartphones and internet technologies among crop farmers around the world with an expected 5.3 billion smartphones by 2025 offers the potential of turning smartphones and the web into a valuable tool for diverse communities growing food. Potential application is the development of web and mobile pest and disease diagnostics through artificial intelligence based-machine learning. Therefore, this paper proposed 24,881 crop disease and pest raw images and 102,976 crop disease and pest augmented images consisting 22 instances. Human society needs to increase food production by an estimated 75% by 2050 to feed an expected population size that is predicted to be over 9.7 billion people [15,16]. Currently, infectious diseases and pests reduce yield by an average of 38% with many farmers in the developing world experiencing yield losses as high as 99%. The increase in the usage of smartphones and internet technologies among crop farmers around the world with an expected 5.3 billion smartphones by 2025 offers the potential of turning smartphones and the web into a valuable tool for diverse communities growing food. Potential application is the development of web and mobile pest and disease diagnostics through artificial intelligence based-machine learning. Therefore, this paper proposed 24,881 crop disease and pest raw images and 102,976 crop disease and pest augmented images consisting 22 instances.

## 2 Data Description

Plant pests and diseases have a negative impact on agricultural production. Delays in discovering plant pests and diseases can generally cause an increase in food insecurity [1]. Early detection helps to prevent and control the plant pest and diseases, which as well play an important role in the management and decision making of agricultural production. Plants infected by pests or diseases show marks or lesions on leaves, stem, flower, or the fruits. Generally, there is a visible pattern that can be used to diagnose any abnormalities of each pest and disease condition. One of the primary sources of identifying plant pests and diseases is the plant leaves, where symptoms of the pest/disease begin to appear [2]. Recently, crop pest and disease detection has been a crucial task and has gained much interest by researchers. This is partly because farmers rely on Extension officers to use their training and experience to diagnose the pests and diseases. This human intervention is not only subjective, but it is also prone to errors, time-consuming, laborious, and inefficient. Less experienced extension officers may provide wrong diagnoses and eventually proffer wrong mitigation measures that may be detrimental to the environment. To alleviate these problems, research into the application of image processing methods for automatic crop pest and disease recognition has become a hot research topic.

Halil et al., 2017 proposed using deep learning algorithms to design a real time detection robot. The study aimed to use the robot to automatically detect plant diseases by moving around in the field or greenhouse. The robot can also detect diseases from close-up photographs taken from plants by sensors built and implemented in the greenhouse [3]. In 2018, Shima et al., adopted Random Forest for classification of healthy and disease leaf from a custom dataset. Their study included several phases of implementation such as data creation, feature extraction, and training models for classification [4]. Muammer et al., 2019 in a study evaluated the performance result of nine deep learning architectures for plant disease detection. In their research a transfer learning coupled with deep feature extraction and deep learning model were used for the disease identification [5]. Lili et al. in 2021 conducted a study to review the progress of deep learning techniques in the area of crop lead disease detection in recent years [6]. The paper presented the current trends and challenges in detecting plant lead disease using deep learning and advanced imaging methods. In [10], a gabor capsule network was proposed for plant disease detection. The experiment of the paper was done using tomato and citrus dataset, which achieved overall accuracy of 98.12% and 98.93%, respectively, using 48 × 48 and 68 × 68 image sizes for the tomato dataset. In addition, on the citrus dataset, the method produced overall accuracy of 93.33% and 92.85%, respectively, using 48 × 48 and 68 × 68 image sizes. In order to improve the model's performance, Mensah et. al., 2020, proposed a gabor capsule network with max-pooling for the detection of plant disease on tomato and citrus datasets [11]. The proposed method demonstrated a better detection of the plant disease compared to the state- of-the-art methods. In [12], Kwabena et. al.  proposed the use of an efficient texture descriptor (Local Binary Pattern -LBP), coupled with sigmoid function, and k-means routing to replace CNN, SoftMax, and dynamic routing in the original capsule network proposed in [13]. The proposed method was evaluated on tomato, maize, and citrus datasets. In [14], a Capsule network with K-Means routing for plant disease recognition using tomato and maize subsets of the PlantVillage dataset and a citrus dataset. The routing algorithm achieved accuracies of 98.80% 97.99% 98.21%, respectively, on the tomato, maize, and citrus dataset.

**Fig. 1:**
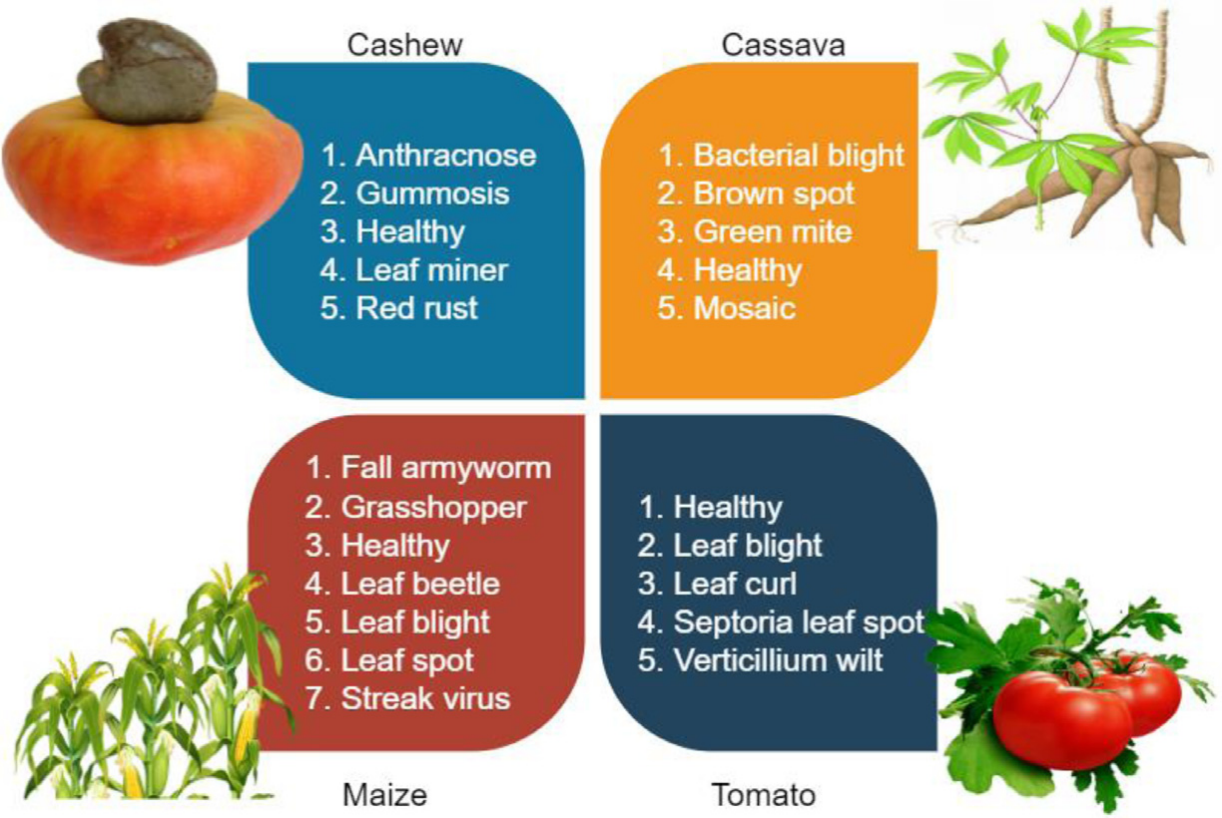
CCMT dataset with their respective classes.

The dataset associated with this paper contains raw (24,881 images) and augmented (102,976 images) color images and consist of twenty-two (22) classes. The images are in varied sizes of (400 × 400), (487 × 1080), (1080 × 518), (3024 × 4032), and (4032 × 3024). This paper provides dataset for deep learning multi-classification, detection, and recognition tasks for single and multiple models. Increasing image resolution for training with deep learning models often has a trade-off with the maximum possible batch size. Yet, the optimal selection of image resolution can further increase neural network performance for various image processing tasks [7]. The Cashew, Cassava, Maize, and Tomato (CCMT) dataset is presented in two folds which are the raw and augmented images. The raw images can be downloaded as a 1.22 GB zip file Raw Data.zip. After unzipping, the Raw data folder contains a subfolder named CCMT Dataset which also contains the Four (4) folders; Cashew, Cassava, Maize and Tomato. Meanwhile, the augmented dataset can be downloaded as a 6.81 GB zip file CCMT Dataset.zip. After unzipping, the main folder CCMT contains the Four (4) folders; Cashew, Cassava, Maize and Tomato. Each of the folders have subfolders. The first folder which is Cashew contains five (5) subfolders; Anthracnose, Gummosis, Healthy, Leaf miner, and Red rust. The second folder, Cassava contains five (5) folders which are Bacterial blight, Brown spot, Green mite, Healthy, and Mosaic. The Maize subfolder also contains seven (7) subfolders namely, Fall armyworm, Grasshopper, Healthy, Leaf beetle, Leaf blight, Leaf spot, and Streak virus. The Tomato subfolder contains five (5) subfolders; Healthy, Leaf blight, Leaf curl, Septoria leaf spot, and Verticillium wilt. Fig. 1 shows the CCMT dataset with the respective classes.

Table 1 shows the specification of the camera used in capturing the dataset. In order to train a deep learning model to identify images under varied conditions (favorable or unfavorable), low quality images as well as their high-quality counterparts are used as input. To achieve this, the camera employed has settings to obtain both types of images. These images were given to experts to label after which a conference of the experts was organized to scrutinize the annotation. The complete dataset is presented and described in Table 2. The table provides details such as the pests and diseases, the direction of image capturing, the background, and the number of images for each class and crop.

**Table 1:** Camera Specifications.

	Description
Camera Name	Canon
Type	EOS Rebel T7
Sensor Type	23.5 × 15.6mm (APS-C) CMOS
Crop factor	1.5x
Shutter Type	Electronic Shutter
Shutter Speed	Electronic Front Curtain Shutter, 1/4000 to 30 Second, 1/4000 to ¼ Second in Movie Mode
Bulb/Time Mode	Bulb Mode
Aspect Ratio	1:1, 3:2, 4:3, 16:9
Image File Format	JPEG, Raw
Image Stabilization	Optical
Optical Zoom	Min 60x
Digital Zoom	4x Minimum (240x Combined Zoom)
Exposure Mode	Aperture Priority, Manual, Program, Shutter Priority
White Balance	Auto, Cloudy, Color Temperature, Daylight, Flash, Incandescent, Shade, white Set 1, White Set 2, White Set 3, White Set 4
Recording Modes	AVCHD/MP4
	UHD 4K (3840 × 2160) at 29.97p
	Full HD (1920 × 1080) at 29.97p/59.94p
	Full HD (1920 × 1080) at 59.94i
	HD (1280 × 480) at 29.97p
	SD (640 × 480) at 29.97p
Recording Limit	Up to 29 Minutes, 59 Seconds
Broadcast Output	NTSC
Auto Recording	Built-In Microphone (Stereo)
Built-In Flash	Yes
Flash Mode	
Effective Flash Range	ISO Auto
External Flash Connection	Intelligent Hot Shoe
Media/Memory Card Slot	Single Slot: SH/SDHC/SDXC
Connectivity	HDMI D (Micro), USB Micro-B (USB 2.0)
Wireless	Wi-Fi
Battery Type	1 x NP-FW50 Rechargeable Lithium-Ion, 1020 VDC
Accessory Mount	1 x Hot Shoe Mount
Focus Type	Auto and Manual Focus
Focus Mode	Continuous-Servo AF, Direct Manual Focus, Manual Focus, Single-Servo AF
ViewFinder	Built-In Electronic
Coverage	100%

**Table 2:** Description of Cashew, Cassava, Maize, Tomato (CCMT) dataset.

S.N.	Crop	Pest / Disease	No. Classes	Direction of image Capturing	Different Backgrounds considered for image capturing	No. of Images of each plant	
						Raw Data	Augmented Data
1	Cashew	Anthracnose	5	Front Direction, Front Direction Rotated 1800,	white, yellow, brown, gray and wild images.	1701	4,940
2		Gummosis				392	2,139
3		Healthy				1,368	7,213
4		Leaf miner				1,358	4,953
5		Red rust				1,682	6,566
6	Cassava	Bacterial blight	5	Backward Direction, Backward Direction Rotated 1800		2,614	8,577
7		Brown spot				1,481	4,734
8		Green mite				1,015	4,266
9		Healthy				1,193	4,569
10		Mosaic				1,205	4,184
11	Maize	Fall armyworm	7			285	1,424
12		Grasshopper				673	3,364
13		Healthy				208	1,041
14		Leaf beetle				948	4,739
15		Leaf blight				1,006	5,029
16		Leaf spot				1,259	5,437
17		Streak virus				1,010	5,049
18	Tomato	Healthy	5			500	2,500
19		Leaf blight				1,301	6,509
20		Leaf curl				518	2,590
21		Septoria leaf spot				2,343	11,715
22		Verticillium wilt				773	3,864
		**Total No. of Images**				**24,881**	**102,976**

**Fig. 2:**
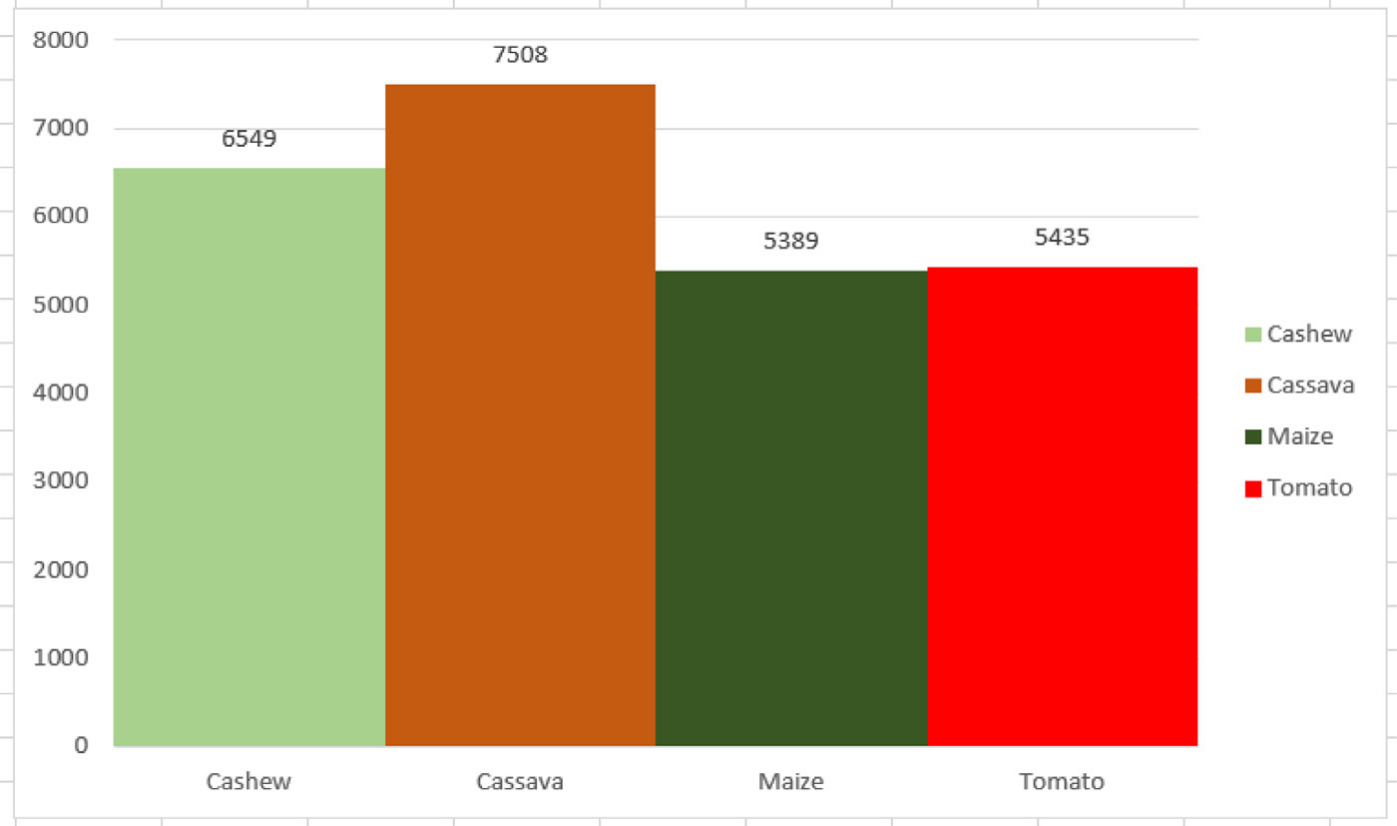
Distribution of the raw CCMT dataset.

**Fig. 3:**
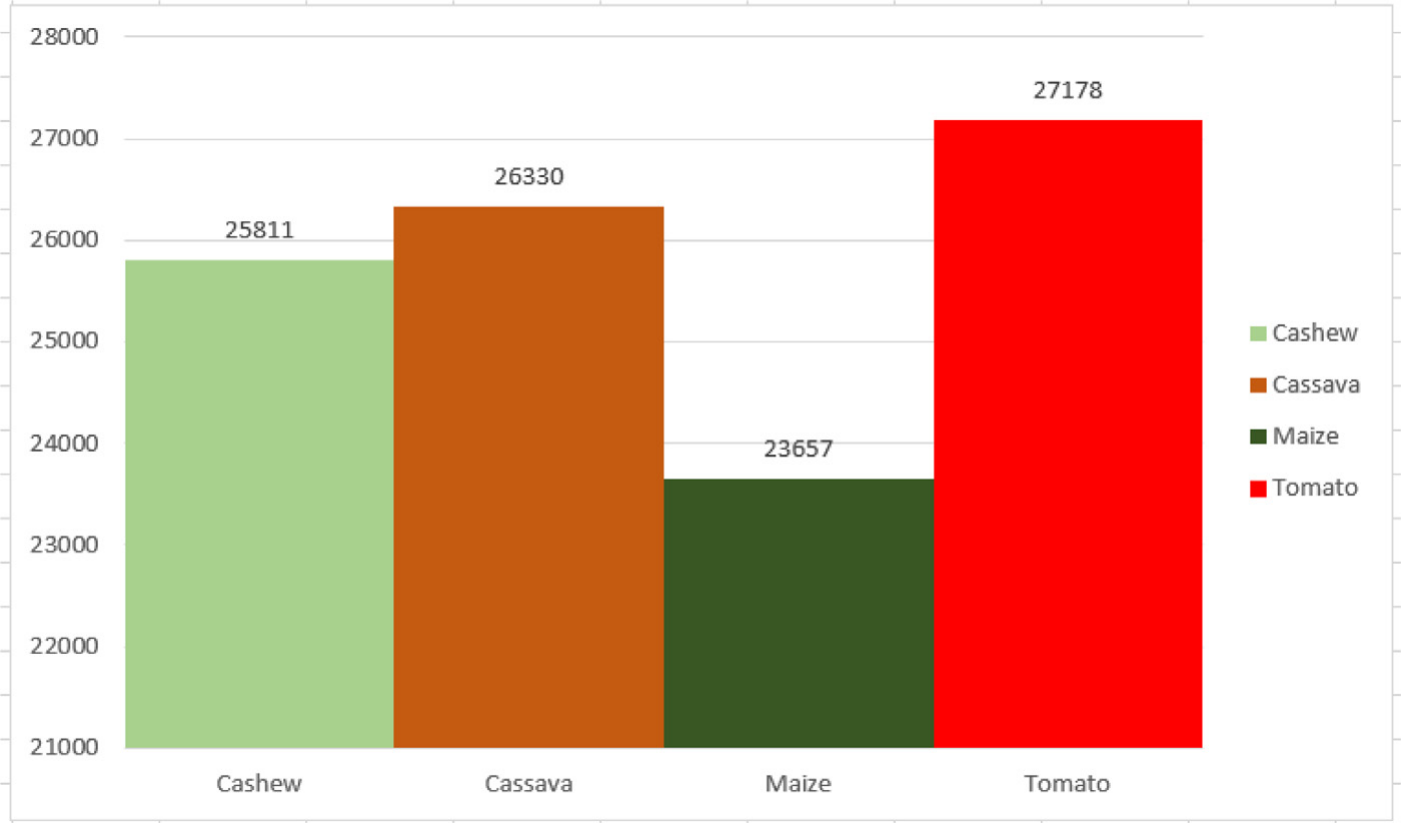
Distribution of the augmented CCMT dataset.

Fig. 2 illustrates the distribution of the raw CCMT dataset. In Fig. 2, it can be observed that the Cashew consists of 6,549 images which represent 26% of the dataset. The Cassava data consists of 7,508 images which is 30% of the total dataset. The Maize consists of 5,389 images representing 22% of the total dataset. Finally, the Tomato data consists of 5,435 images comprising 22% of the total dataset. In order to improve the generazability of the model, the raw images were augmented to increase the size of the dataset. Fig. 3 presents the distribution of the augmented CCMT dataset. It can be observed that the Cashew consists of 25,811 images which represent 25% of the dataset. The Cassava data consists of 26,330 images which is 26% of the total dataset. The Maize consists of 23,657 images representing 23% of the total dataset. Finally, the Tomato data consists of 27,178 images comprising 26% of the total dataset.

**Fig. 4:**
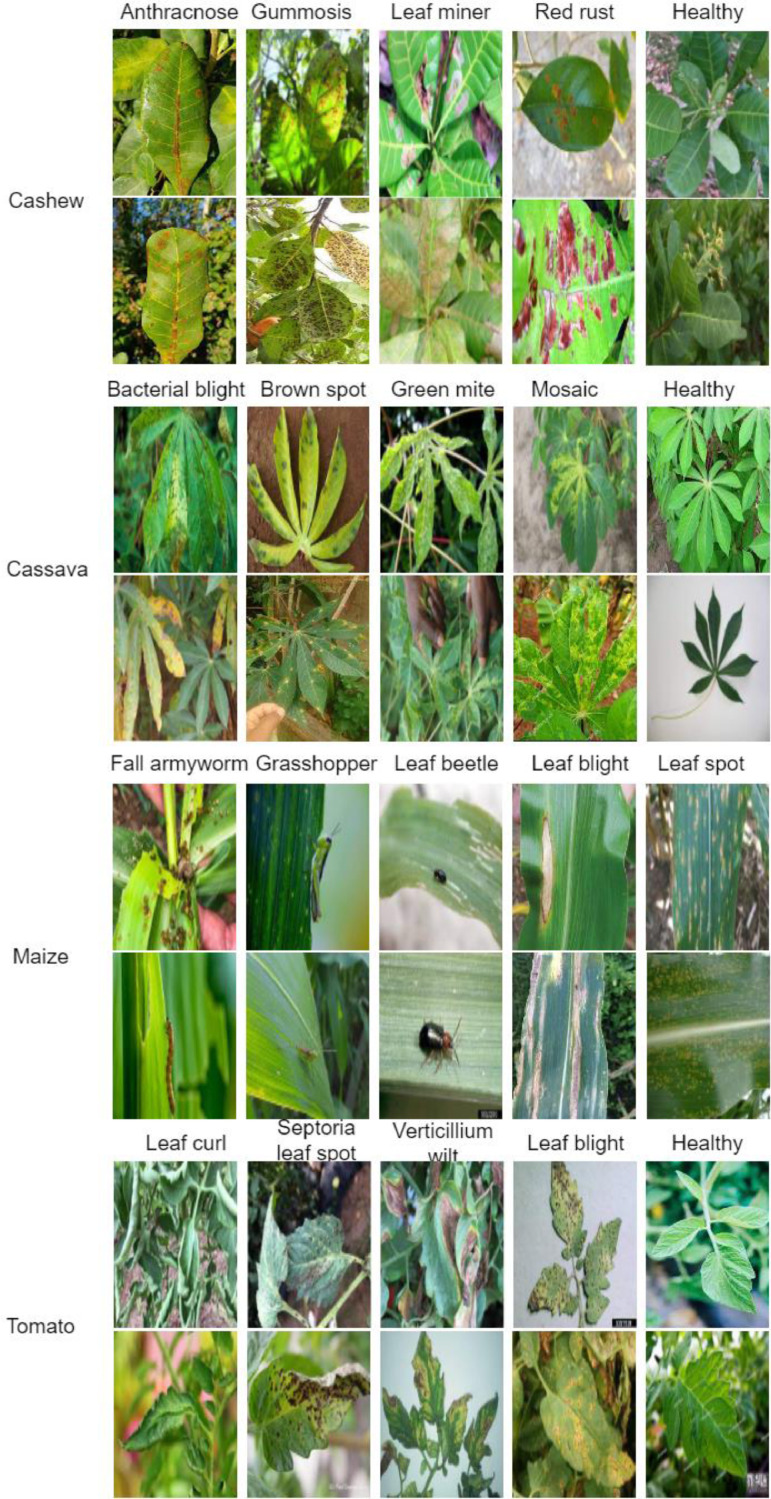
Data Samples of the CCMT dataset images.

**Fig. 5:**
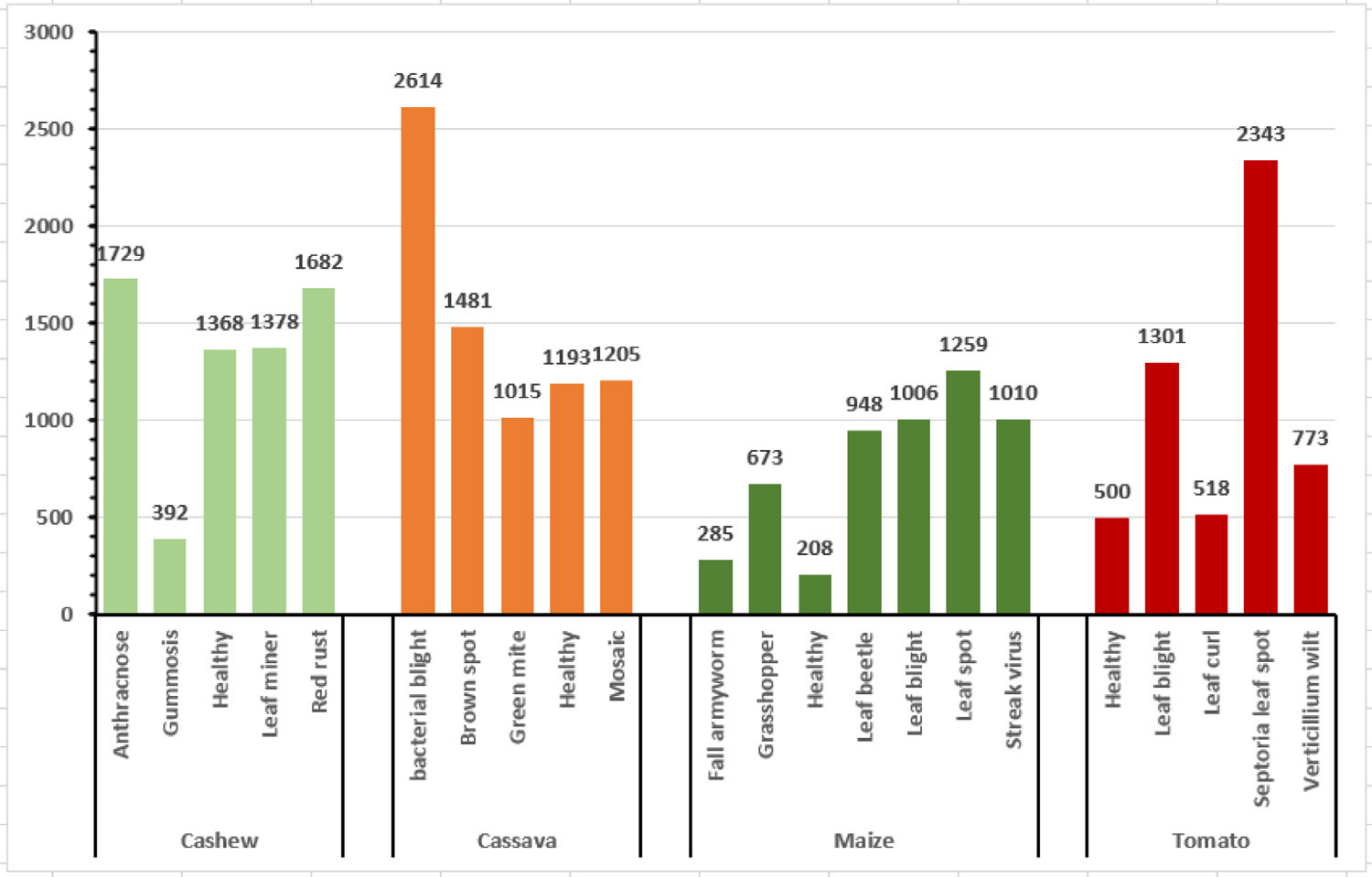
Pest and Disease in the raw CCMT dataset.

**Fig. 6:**
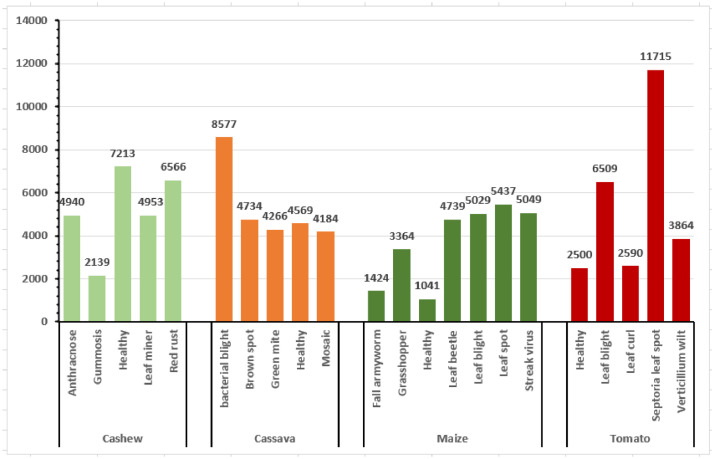
Pest and Disease in the augmented CCMT dataset.

**Fig. 7:**
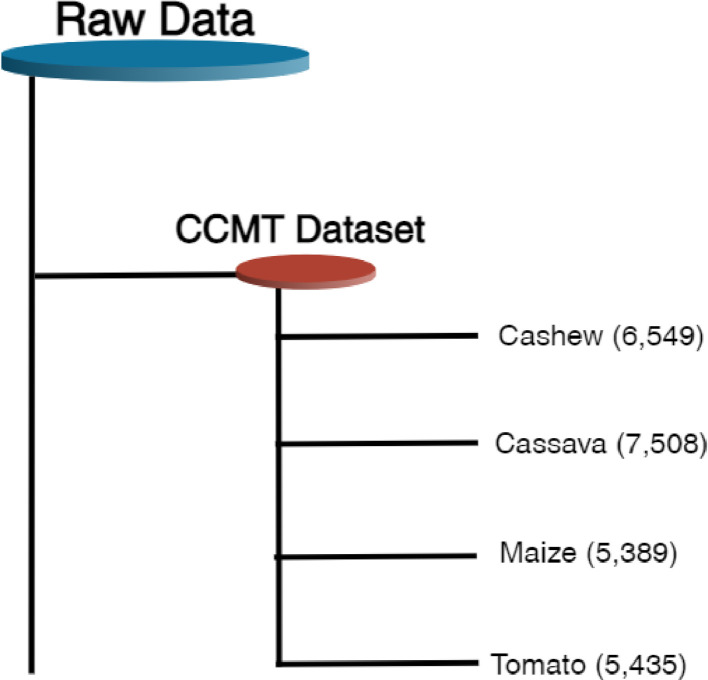
Directory structure of the raw CCMT dataset.

Fig. 4 presents some data samples of the CCMT images from the various classes of the respective crops. The samples show both the crops and pests/diseases affecting each crop. Figs. 5 and 6 presents the pest and disease images in the raw and augmented datasets. The directory structure of the dataset is shown in Fig. 7. The directory describes the folder structure of the CCMT dataset. The first folder is CCMT which contains subfolders named Cashew, Cassava, Maize and Tomato.

## 3 Experimental Design, Materials and Methods

### 3.1 Experimental Design

**Fig. 8:**
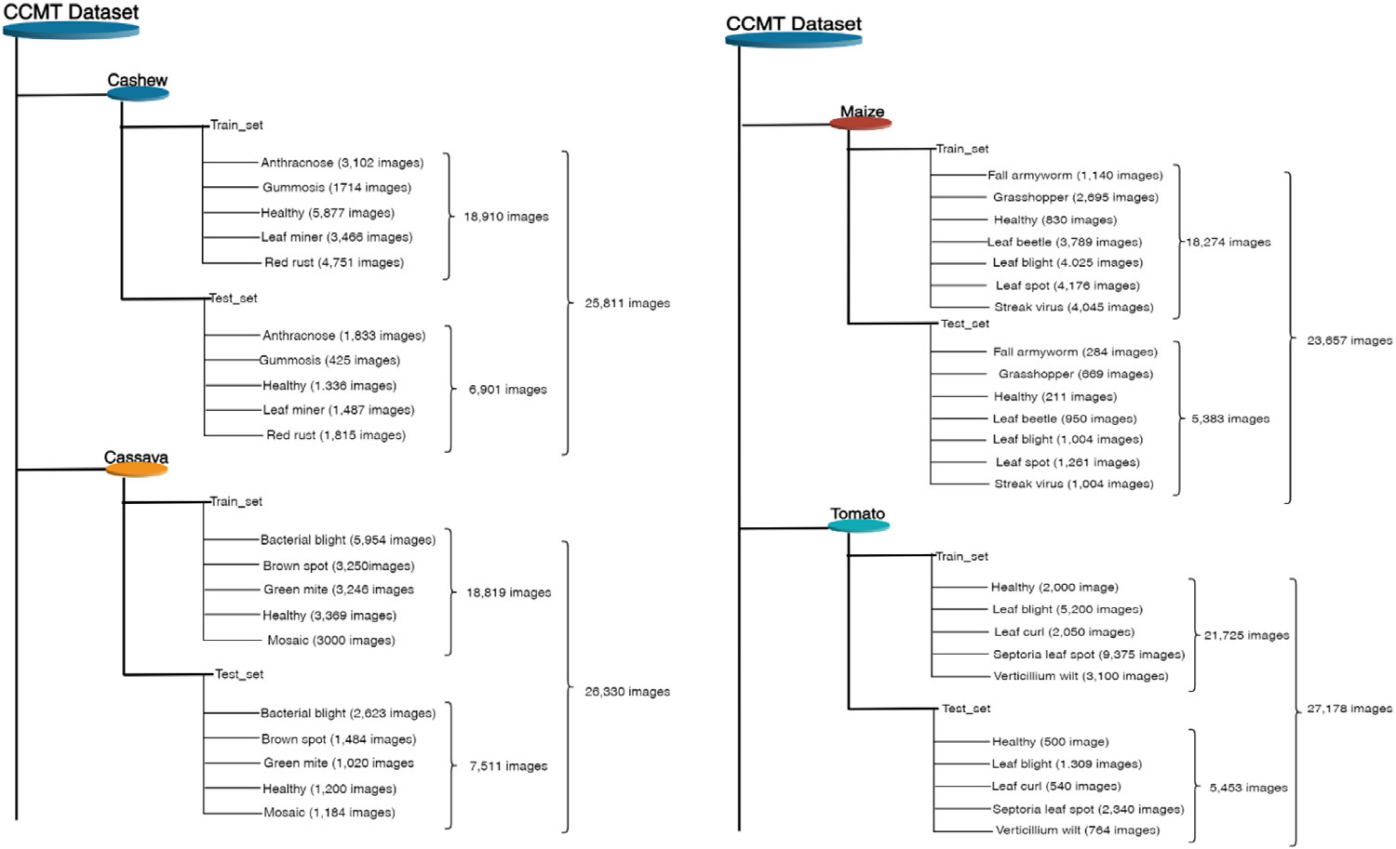
Directory structure of the augmented CCMT dataset.

**Fig. 9:**
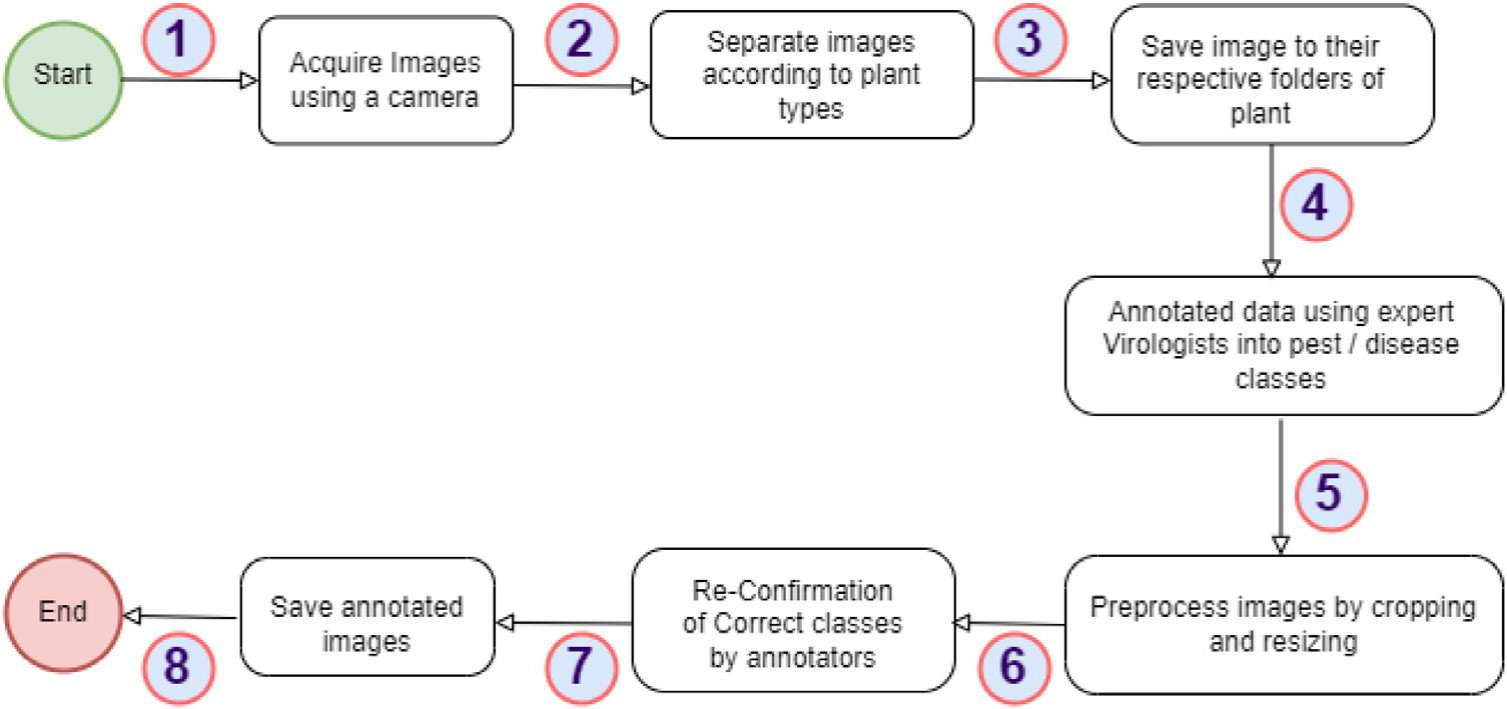
The CCMT dataset acquisition process.

Fig. 9 shows the image data acquisition process. The images were captured using a Canon EOS Rebel T7 DSLR EF-A 18-55mm high-resolution rear camera. All images were captured, separated, and saved in their respective folders according to the plant type. The images were annotated using expert annotators made up of plant virologists and pathologists. Each expert was given parts of the dataset depending on their area of expertise and a period of three weeks for the annotation and labelling. Subsequently, each two experts exchanged data to verify the authenticity of the labeling. All the experts were then called to a conference for a thorough discussion of the annotation/labeling. Images for which there was no consensus were eliminated from the dataset. The annotated images were saved in their respective folders and preprocessing carried out. The preprocessing included image cropping to focus on the regions of interest (ROI), or area lesions on the leaf. Table 3 presents the timelines of the dataset acquisition process which took place during the lean crop season in Ghana. The images were captured daily and during the day time from October 2022 to December 2022. The images were captured in different directions and backgrounds and with varied sizes, as specified in Table 2. The folder structure of the images is shown in Figs. 7 and 8. The dataset consists of only sick, healthy and pests of the four crops.

**Table 3:** Data acquisition steps.

No.	Step	Duration	Activity
1	Data Gathering	October 2022 to December 2022	Daily and during daytime capturing of the plant images
2	Image Annotation	December 2022 to January 2023	Labeled the 24,916 images of Plant pest and disease images
3	Image Preprocessing	January 2023	Cropping and size reduction of the plant data

### 3.2 Materials or specifications of the image acquisition system

The Cashew, Cassava, Maize, and Tomato (CCMT) images were captured using Canon EOS Rebel T7 DSLR EF-A 18-55mm Lens camera. All the original image datasets are of varied sizes (400 × 400), (487 × 1080), (1080 × 518), (3024 × 4032), and (4032 × 3024) and are saved in .jpg format.

Computer vision algorithms can learn and generalize efficiently if only they are provided with input images sourced under different conditions such as varied background and illumination changes. Illumination of the scene is a factor under the control of the environment and the quality of the image sensor of the capturing device [8]. The introduction of different lighting conditions can help alleviate the reduction in performance [9] of deep learning models. Consequently, the CCMT images are captured under various environmental conditions backgrounds, and angles as stipulated above.

## Ethics Statements

The dataset that supports this work was collected from farms where farmers agreed for the collection. A consent form to seek approval for the data collection was presented and filled by farmers given their approval to embark on the data collection in their various farms.

## CRediT authorship contribution statement

**Patrick Kwabena Mensah:** Data curation, Conceptualization, Supervision. **Vivian Akoto-Adjepong:** Methodology, Software, Writing – original draft. **Kwabena Adu:** Methodology, Software, Writing – original draft. **Mighty Abra Ayidzoe:** Methodology, Software, Writing – original draft. **Elvis Asare Bediako:** Data curation, Validation. **Owusu Nyarko-Boateng:** Data curation, Conceptualization, Supervision. **Samuel Boateng:** Writing – review & editing. **Esther Fobi Donkor:** Data curation, Validation. **Faiza Umar Bawah:** Data curation, Conceptualization, Supervision. **Nicodemus Songose Awarayi:** Data curation, Conceptualization, Supervision. **Peter Nimbe:** Writing – review & editing. **Isaac Kofi Nti:** Writing – review & editing. **Muntala Abdulai:** Data curation, Validation. **Remember Roger Adjei:** Data curation, Investigation. **Michael Opoku:** Data curation, Investigation. **Suweidu Abdulai:** Data curation, Investigation. **Fred Amu-Mensah:** Writing – review & editing.

## Declaration of Competing Interest

The authors declare that they have no known competing financial interests or personal relationships that could have appeared to influence the work reported in this paper.

## Data Availability

Dataset for Crop Pest and Disease Detection (Original data) (Mendeley Data).
